# Oxyhemoglobin Concentration and Oxygen Uptake Signal During Recovery From Exhaustive Exercise in Healthy Subjects—Relationship With Aerobic Capacity

**DOI:** 10.3389/fphys.2021.695569

**Published:** 2021-07-02

**Authors:** Małgorzata Żebrowska, Matthias Weippert, Monika Petelczyc

**Affiliations:** ^1^Faculty of Physics, Warsaw University of Technology, Warsaw, Poland; ^2^Institute of Sport Science, University of Rostock, Rostock, Germany

**Keywords:** recovery phase, physical effort, oxygen uptake, oxyhemoglobin concentration, Hill’s model, exponential model

## Abstract

This proof of concept study is dedicated to the quantification of the short-term recovery phase of the muscle oxygenation and whole-body oxygen uptake kinetics following an exhaustive cycling protocol. Data of 15 healthy young participants (age 26.1 ± 2.8 years, peak oxygen uptake 54.1 ± 5.1 mL^∗^min-1^∗^kg-1) were recorded during 5 min cool down-cycling with a power output of 50 W on an electro-magnetically braked cycle ergometer. The oxygen uptake (VO_2_) signal during recovery was modeled by exponential function. Using the model parameters, the time (T_1/2_) needed to return VO_2_ to 50% of VO_2__peak_ was determined. The Hill’s model was used to analyze the kinetics of oxyhemoglobin concentration (Sm, %), non-invasively recorded by near-infrared spectroscopy (NIRS) over the M. vastus lateralis. Analysis of the Pearson correlation results in statistically significant negative relationships between T_1/2_ and relative VO_2__peak_ (*r* = −0.7). Relevant significant correlations were determined between constant defining the slope of VO_2_ decrease (parameter B) and the duration of the anaerobic phase (*r* = −0.59), as well as between Hill’s coefficient and average median Sm_max_ for the final 2 min of recovery. The high correlation between traditional variables commonly used to represent the cardio-metabolic capacity and the parameters of fits from exponential and Hill models attests the validity of our approach. Thus, proposed descriptors, derived from non-invasive NIRS monitoring during recovery, seem to reflect aerobic capacity. However, the practical usefulness of such modeling for clinical or other vulnerable populations has to be explored in studies using alternative testing protocols.

## Introduction

Cardiopulmonary exercise testing (CPET) is a non-invasive standard clinical tool used to investigate exercise tolerance, diagnose pathophysiological mechanisms within the cardiovascular-metabolic system, to define treatment options as well as to predict their potential outcomes (Albouaini et al., 2007; Takken et al., 2019). Among the CPET methods, the ramp incremental test is commonly used to assess physical fitness and/or the limitations set by the cardio-vascular system under standardized laboratory conditions (Michalik et al., 2019). In clinical practice diagnostic information is obtained from the physiological response at volitional exhaustion, i.e., peak (VO_2__peak_) oxygen uptake (Day et al., 1985), peak power output, maximal heart rate and peak blood lactate (Midgley et al., 2007; Poole et al., 2008). However the kinetics of measured signals during submaximal efforts might have the potential to evaluate the degree and change of physical fitness, exercise tolerance and to elucidate pathophysiological mechanism to the same extent as classical markers (Laveneziana et al., 2021). Thus, it has been recently shown that NIRS-derived markers of skeletal muscle respiration are better predictors of time-trial performance in athletes than traditional markers of endurance performance using an exhaustive cycling test (Batterson et al., 2020).

NIRS has improved the understanding of skeletal muscle physiology since the end of the 1980s. In principle, NIRS uses the differing light absorption of oxygenated and deoxygenated hemoglobin/myoglobin (Grassi and Quaresima, 2016). Different measurement and analysis techniques have evolved that enable the estimation of skeletal muscle oxygen kinetics not only in scientific research under standardized laboratory condition but also for the application in the field, daily exercise and practice (Ferrari et al., 2011). Further, in the last decade protocols for estimating oxidative capacity of the skeletal muscle utilizing NIRS have been developed and validated. These techniques are well suited to monitor changes in muscle specific aerobic adaptations over time and thus, may enable the evaluation of the effectiveness of interventions at the muscular level (Hanna et al., 2021). Further, associations between the oxidative metabolism of the gastrocnemius muscle, utilizing NIRS during an arterial occlusion protocol, and whole-body VO_2__peak_ have been shown (Lagerwaard et al., 2019).

These and other findings (e.g., Soller et al., 2008) let us assume that indices of muscular O_2_-kinetics during recovery from a standard exercise stimulus may have the potential to estimate whole body aerobic capacity, i.e., VO_2__peak_, as this parameter combines both peripheral O_2_-utilization by the working muscles and central aspects of O_2_-delivery.

Continuous wave-based NIRS systems were developed to measure the saturation of the muscle with oxygenated hemoglobin (Sm, %), whereby Sm, reflects the dynamic balance between muscular O_2_-supply and -consumption (Ferrari et al., 2011). Because they are relatively low cost and highly portable, they provide a range of potential applications including not only the estimation of peripheral O_2_-kinetics but in combination with standardized load protocols also information on aerobic metabolism and capacity.

Currently, CPET to volitional exhaustion is the gold standard to assess physical fitness in terms of maximal VO_2_. However, there exist some problems in the clinical and scientific practice. Often, it is difficult to determine a valid value due to the fact that in many subjects the required VO_2_-plateau is not attainable and many clinicians and researchers are concerned about excessively straining their subjects during heavy exercise, particularly those with chronic disease and conditions like obesity. Thus, potentially predictive exercise biomarkers, such as exercise onset, recovery gas exchange kinetics, and heart rate variables might be of practical diagnostic value (Belfry et al., 2012; Mann et al., 2014; Orellana et al., 2019). Recovery (REC) is known as the post-exercise process of returning metabolism to its pre-exercise state (Tomlin and Wenger, 2001). Usually, it is distinguished into two stages: an initial rapid phase, lasting from 10 s to several minutes and a slower one, lasting from a few minutes to several hours. During the first phase of regeneration, heart rate and the level of oxygen consumption decrease rapidly (Tomlin and Wenger, 2001). This process is associated with the rapid replenishment of oxygen reserves in the tissues and the restoration of most of the ATP and phosphocreatine, depleted during physical exertion (Gaesser and Brooks, 1984). The slower REC component is characterized by removal of lactate and H+, elevated body temperature, ventilation, and circulation (Gladden et al., 1982). The O_2_-kinetics may further reflect a substrate shift toward fat oxidation, triglyceride, and fatty acid cycling (Børsheim and Bahr, 2003).

Due to the complexity of physiological changes during REC, there is a need for considering kinetics and mathematical models, quantitatively describing the physiological signals. We hypothesize the usefulness of the exponential and Hill model in the description of the early phase of recovery after incremental effort until exhaustion in a healthy people group. Using the parameters determined for both models (in oxygen uptake and oxyhemoglobin concentration), we investigated their correlations with standard aerobic capacity descriptors. In case of significant and high correlations, the approach has to be validated using alternative submaximal testing protocols to prove its usefulness for its application in clinical and other populations of interest.

## Materials and Methods

### Participants

The data used in this work was recorded at the Institute of Sports Sciences of the University of Rostock (Germany). Fifteen healthy and young men (26.1 ± 2.8 years), with mean VO_2__peak_ value 54.1 ± 5.1 mL/min/kg and body mass index 23.6 ± 1.8 kg/m^2^ participated in the study. All participants were free of medication and abstained from any exhaustive exercise and alcohol for > 48 h prior to the experiment. Furthermore, the consumption of caffeine or nicotine was not allowed during the night and on the morning of the experiment. After a medical clearance participants performed an incremental cycling test until volitional exhaustion. All participants were highly motivated, additionally strong verbal encouragement was given during the test. The study was approved by the local ethics committee (registration no. A 2017-0034) at the University of Rostock.

### Protocol

The experiment protocol was divided into: (1) 6 min adaptation (REST) without load; (2) 3 min Baseline phase at 50 W; (3) phase of load increments by 25 W / min until volitional exhaustion; (4) 5 min recovery (REC) phase at 50 W load. In the study, the SRP 3000 bicycle ergometer (Sportplus, Germany) was used, which allows for systematic increase of the load. The MetaMax 3B system (Cortex Biophysics Inc., Germany) was used to record the cardiopulmonary response. The signals were measured by breath by breath technique. Simultaneously the oxyhemoglobin concentration in the working quadriceps muscle was recorded by Moxy Monitor (Fortiori Design LLC, Hutchinson, MN, United States). The Moxy Monitor has been developed for the practical application in sports and exercise, providing an accuracy useful for sport science applications. It has recently been tested in terms of validity and reliability (see Feldmann et al., 2019 for detailed technical information) and provides the Sm of the muscle of interest on an *a priori* scale from 0 to 100%. Two detectors, spaced 12.5 and 25 mm from the NIR light source, measure the amount of light emitted at four wavelengths in a diffuse reflectance configuration, leading to a total of eight measurements. The default sampling across the four wavelengths is done at a rate of 80 per 2 s, whereby the averages are reported at 0.5 Hz (Feldmann et al., 2019).

For the measurements the NIRS device had been placed over the right M. vastus lateralis, halfway between the greater trochanter and the lateral epicondyle of the femur. Skin below the NIRS device was shaved and cleaned. The transmitter was then fixed and shielded from ambient light using elastic bandages and a cover supplied by the manufacturer. This type of fixation also prevented the sensor from moving on the skin during exercise.

From the recorded experimental data the following parameters were obtained: VO_2__peak_, T_1/2_—time needed for the oxygen uptake value to return to 50% of the peak value after the maximum load ceases, P_max_—the maximum power output during the trial, T_anaerobic_—duration of the anaerobic phase (time duration between second—VT2 and first ventilatory thresholds, VT1). VT1 was obtained using the V-slope method (Beaver et al., 1985). VT2 was determined as the first crossover in the relation of minute ventilation and carbon dioxide output.

To infer the occurrence of characteristic phenomena in the signal from the REC, we referred to the signal from the baseline phase. In both, the baseline and REC, participants cycled at the same load (50 W). Due to the lagging VO_2_-uptake kinetics during warm-up, the initial part of experimental protocol was excluded from the analysis and only the last 2 min of baseline were considered as reference for REC cycling. In further parts of the work, the following terms were denoted:

BAS—the last 2 min from baseline
RecBegin—the initial 2 min from REC
RecEnd—the final 2 min from REC.

### Data Analysis

The methodological approaches and procedures are described below.

#### Preprocessing

**FIGURE 1:**
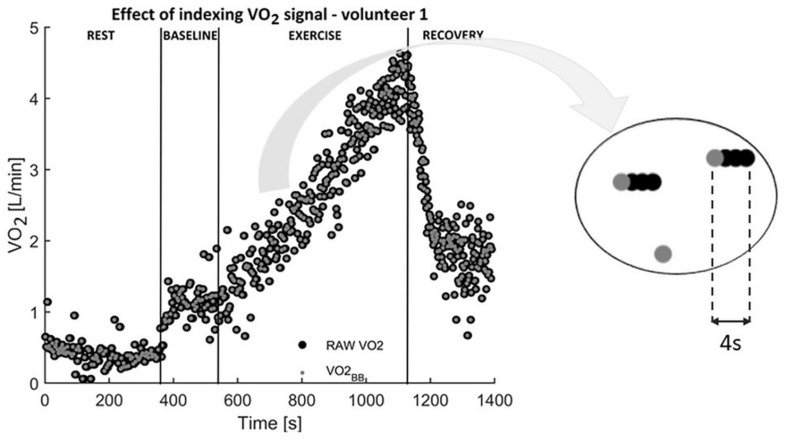
Original oxygen uptake signal (black) was limited to only the first recorded VO_2_ value of each breath. As a result, a new VO_2_BB_ signal sampled irregularly (gray) was obtained.

Due to the breath-by-breath technique used in the measurement of ventilatory data, the oxygen uptake signal was characterized by the same successive VO_2_ variables. In order to avoid repeated recorded values, the raw VO_2_ signal (sampled at 1 Hz) was resampled by dedicated procedure. For the further analysis the data was limited to the first value of each breath, causing that only one measurement point was given per breath. The total number of measurement points was reduced and a non-uniformly sampled signal VO_2_BB_ was obtained (Figure 1). Using proposed method of preprocessing, we excluded stepwise fluctuations in construction of the model fit.

#### Exponential Model for Recovery Oxygen Uptake Curve

In order to quantitatively compare the properties of oxygen uptake in the group of healthy subjects, an exponential model was constructed to estimate the kinetics of the VO_2_ after the termination point of the incremental load. Previous studies (Cohen-Solal et al., 1995; Vianna et al., 2014) have proven the diagnostic value of exponential model parameters. In the study (Cohen-Solal et al., 1995), it was shown that the kinetics of the return of oxygen uptake to resting values after exercise is prolonged in parallel with the recovery of muscle energy reserves. In Vianna et al. (2014), authors reported that in healthy and trained subjects, VO_2_ kinetics depend on muscle performance and is not limited to a large extent by the efficiency of the O_2_-transport system. Therefore, the Eq. (1) was chosen:

(1) $$V\,O_{2}\,\left(t\right)=A^{*}\,e^{-t\,B}+C$$

where: A (L/min)—oxygen uptake value during maximum effort, B (s)—constant defining the slope of oxygen uptake decrease, C (L/min)—oxygen uptake value in the final stage of recorded REC phase.

Parameter A represents the oxygen uptake value in the final stage of maximum load occurrence. Parameter B is related to the VO_2_ kinetics during REC. Parameter C represents the exponential curve shift parallel to the *x*-axis. It reflects a stabilization of the oxygen uptake value in the short term recovery. Non-linear regression was performed in Origin, 9.0 using the Levenberg-Marquardt (LM) least square non-linear regression algorithm, which is typically used in case of curve-fitting problems. The LM procedure relies on the minimalization of the sum of the squares of the distances between the model function and the set of data points. The reduction of the sum of the squares is performed in iterative manner by “updates” to model parameters. In LM algorithm, two other minimalization procedures are merged to find fast the best approximation for model parameters. Detailed description of the LM method can be found in Gavin (2020).

#### Hill’s Model for Oxyhemoglobin Concentration During Recovery

To model the oxyhemoglobin concentration kinetics, we decided to use the equation developed in 1910 by Archibald Hill, an English physiologist and Nobel laureate (Hill, 1921). Based on the observations of the binding of oxygen to hemoglobin and the assumption that the cooperativity arises as a result of aggregation of hemoglobin molecules, each of which binds one oxygen molecule, the scientist proposed the first expression for cooperative binding to this multisite protein (Stefan and Le Novère, 2013).

The basic expression called the Hill equation is as follows:

(2) $$S\,m\,\left(t\right)=S\,m_{m\,a\,x}\,\frac{t^{n}}{\left(k^{n}+t^{n}\right)}$$

Where Sm_max_ (%) is related to the maximum percentage of saturated oxyhemoglobin concentration, k (s)—time needed for hemoglobin concentration to return to 50% of the maximum value, n (no unit)—Hill’s coefficient.

A Hill’s coefficient n > 1 indicates positive cooperativity binding of oxygen to hemoglobin. If n < 1, the system exhibits negative cooperativity (Stefan and Le Novère, 2013). The Hill’s coefficient equal to 1 reflects none cooperativity for a monomeric protein like myoglobin. Molecular binding is called an interaction between molecules that results in a stable chemical association between them. When the number of macromolecular binding sites occupied by a particular type of ligand is a non-linear function of the concentration of this ligand, it is said that a cooperative binding has occurred (Stefan and Le Novère, 2013). Positive cooperativity occurs when ligand binding increases affinity to other sites (finding one ligand makes it easier for a protein to bind a second, third…). In contrast, negative cooperativity results in decreased affinity. In addition to Hill’s original approach, in which the model was used to analyze oxygen binding by hemoglobin (Hill, 1910), Eq. 2 was applied to the analysis of enzymatic reactions (Michaelis et al., 2011) and fluctuations of acetylcholine concentration in muscle cells (Benfey and Grillo, 1963). In pharmacology, the Hill’s equation has been proposed to describe the relationship between the concentration of drug and the intensity of a pharmacologic response (Wagner, 1968). Archibald Hill also discussed the energy related to electric changes in muscles and nerves (Hill, 1921).

## Results

### Recovery Oxygen Uptake Analysis

**FIGURE 2:**
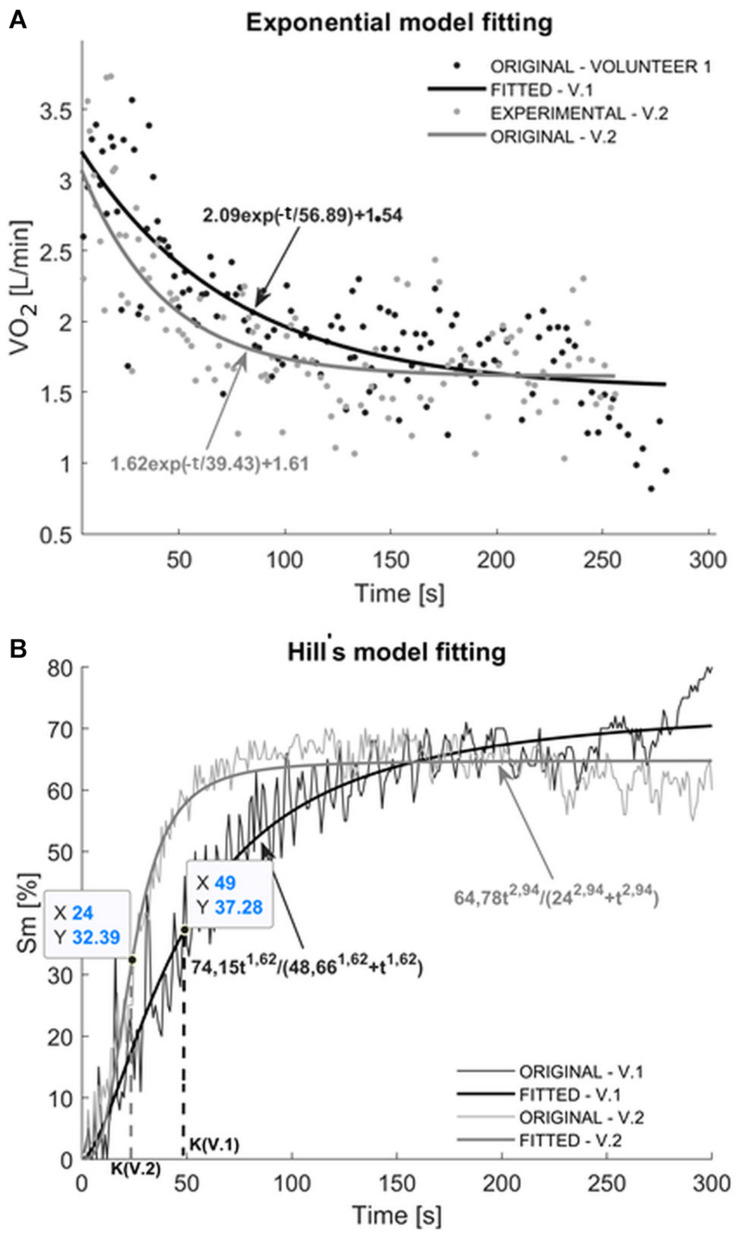
**(A)** Example of oxygen uptake (VO_2_) during the REC phase in two volunteers (V.1 and V.2) with fitted theoretical values derived from the exponential model. **(B)** Effect of fitting the Hill’s model to the experimental oxyhemoglobin concentration (Sm) data during the recovery for two study participants.

The effect of fitting the exponential function to VO_2_ data records from REC is presented in Figure 2A. Despite the similar decreasing trend of oxygen consumption for Volunteer 1 (V.1) and 2 (V.2), the estimated coefficients of the model (Eq. 1) differ from each other. The curves presented in Figure 2A have also different starting values (VO_2_ = 2.09 L/min) for V.1 and (VO_2_ = 1.62 L/min) for V.2, respectively. A higher value of parameter B was obtained for V.1 (56.89 s) than for V.2 (39.43 s). B reflects the decline of the exponential curve in the initial phase of recovery (approximately 2 min). Note, the higher the B coefficient value, the slower the decrease of the VO_2_.

**FIGURE 3:**
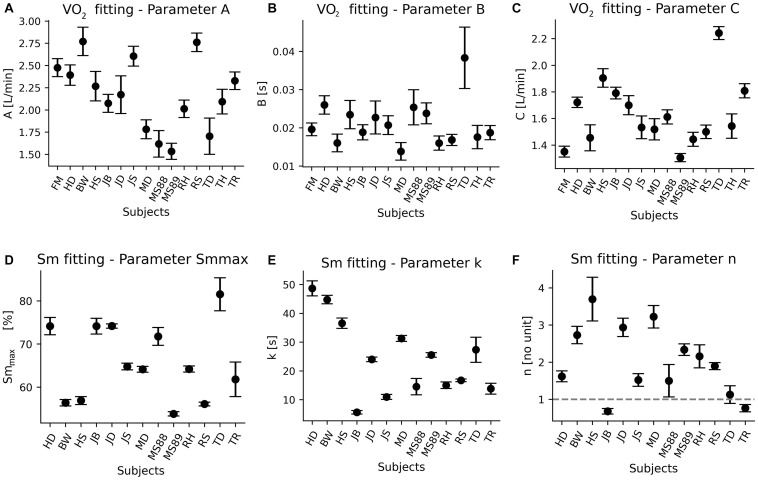
Values of estimated parameters and 95% confidence intervals obtained from oxygen uptake (VO_2_) and percentage oxyhemoglobin concentration (Sm) signals in the group of healthy subjects. 95% confidence interval was calculated on the basis of the standard error value in accordance with the relationship: 1.96 * standard error. Values of the A **(A)**, B **(B),** and C **(C)** coefficients are determined from exponential model (Eq. 1) and Sm_max_ **(D)**, k **(E)**, n **(F)** are estimated from Hill’s equation (Eq. 2).

The determined values of parameter A in the analyzed group of healthy subjects vary in the range: 1.53–2.77 L/min. The estimated parameter B changes from 26.09 to 72.31 s. Whereas parameter C belongs to the range of 1.35–2.24 L/min. To assess the uncertainty of the determined parameters, a comparison of their 95% confidence intervals was considered and presented in Figures 3A–C by errorbars. Intervals were obtained by calculating the percentage difference between the upper and lower limit values. Results indicate that parameter B is determined the least accurate. The widths of the uncertainty vary from 39.70% to even 118.91%. The parameter A was estimated with the uncertainty estimated to the range 15.29–59.94 %. With the greatest accuracy exponential algorithm determined the C parameter (9.15–31.26%). The quality of exponential model fitting was also quantified by the coefficient of determination *R*^2^. We observed clearly lower (*R*^2^ < 0.5) for two subjects. For other participants, the parameter *R*^2^ ranges from 0.57 to 0.85.

### Recovery Oxyhemoglobin Concentration Analysis

Figure 2B presents an example of fitting the Hill’s model (according to Eq. 2) to the percentage oxyhemoglobin concentration recorded during REC. The signal obtained after the termination of the load is characterized by a rapid increase of oxyhemoglobin level until the plateau phase, where Sm exhibits only minor fluctuations as REC continues. In the examples presented in Figure 2B, obtained from the model values of the Sm_max_ are 74.15% for V.1 and 64.78% for V.2. The k parameter reflects the time needed to return to half the Sm_max_. The k parameters estimated for V.1 and V.2 are equal 48.66 and 24 s, respectively. The time periods were determined also directly from records (K parameter marked in Figure 2B). The V.1 takes approximately 49 s to return to 50% maximal oxyhemoglobin concentration, while V.2 reaches 50% Sm_max_ just after 24 s. It can be concluded that the second subject is characterized by a faster return of the oxyhemoglobin level similar to BAS values. This is also observed in the shapes of the curves at the initial 50 s of the REC phase. The values of the n coefficients are greater than 1, which, in accordance with the previously presented theory of parameter interpretation, proves the positive cooperative binding of oxygen with hemoglobin.

Due to two missing records, 13 oxyhemoglobin concentration datasets from the initial group of 15 participants were analyzed. Figures 3D–F presents the values of the estimated parameters Sm_max_, k, and n with the uncertainties specified by 95% confidence intervals. In the studied group, the maximum oxyhemoglobin concentration Sm_max_ obtained during the REC phase varies in the range of 53.8–89.13% (Figure 3D). Parameter k indicating the increase of oxygenated hemoglobin during REC after maximal load is equal from 5.61 to 48.66 s (Figure 3E), while the Hill’s index n varies from 0.68 to 3.22 (Figure 3F). Again, the assessment of the accuracy of the parameters was performed and presented in the form of errorbars. The Sm_max_ parameter was determined with the highest accuracy, the 95% confidence intervals varies from 1.11 to 13.18%. The uncertainty of determining the parameter k changes from 5.79 to 49.05%. The widest confidence intervals (10.94–80.68%) were recorded for the n parameter.

### Correlations of Model Parameters With Traditional CPET Measures

The values of the correlation between the maximum load, classical endurance parameters and the measures obtained from the models of REC phase were determined. The estimated coefficients of the exponential model for the VO_2_ signal (Table 1) and the Hill’s model for the percentage concentration of Sm (Table 2) were compared with the traditional markers of CPET.

**TABLE 1:** Linear Pearson correlation coefficients for the model parameters of oxygen uptake kinetics and traditional markers of cardiorespiratory fitness.

	T_1/2_ (s)	Relative VO_2peak_ (mL/kg/min)	P_max_ (W)	T_anaerobic_ (s)
A (L/min)	−0.12	0.27	0.37	−0.19
	*p* = 0.677	*p* = 0.337	*p* = 0.174	*p* = 0.489
B (s)	**0.61**	−0.24	−0.12	**−0.59**
	*p* = 0.016*	*p* = 0.398	*p* = 0.659	*p* = 0.020*
C (L/min)	−0.50	**0.77**	0.27	0.43
	*p* = 0.060	*p* = 8.549*10^–4^*	*p* = 0.329	*p* = 0.111
T_1/2_ (s)	–	**−0.70**	−0.16	−0.01
		*p* = 0.005*	*p* = 0.576	*p* = 0.971

*p < 0.05. Bold values indicate Pearson correlation coefficient with statistical significance <0.05.

**TABLE 2:** Values of linear Pearson correlation for Hill model parameters and markers characterizing the respiratory and muscular responses to the physical activity phases.

	T_1/2_ (s)	Relative VO_2peak_ (mL/kg/min)	Median of Sm (%)	
			RecBegin	RecEnd
Sm_max_ (%)	−0.38	0.45	0.14	0.72
	*p* = 0.284	*p* = 0.223	*p* = 0.675	*p* = 0.998
k (s)	0.35	−0.03	0.13	0.70
	*p* = 0.322	*p* = 0.931	*p* = 0.659	*p* = 0.997
n (no unit)	0.26	−0.36	0.13	**−0.52**
	*p* = 0.466	*p* = 0.304	*p* = 0.659	*p* = 0.033*

*p < 0.05. Bold values indicate Pearson correlation coefficient with statistical significance <0.05.

According to Table 1, we have observed a high negative Pearson correlation (*r* = −0.70, *p* = 0.005) between the T_1/2_ parameter and VO_2__peak_. It is similar to previously published results (Hargreaves and Spriet, 2020). The higher the VO_2__peak_, the shorter time needed for oxygen uptake to return to 50% of the peak value. Further, a relationship between the time constant B, describing the decline of the exponential curve in the initial REC phase, and T_anaerobic_ was evident. The negative correlation coefficient (*r* = −0.59, *p* = 0.020) indicates that in the group of tested subjects, the longer the duration of the anaerobic phase, the faster reduction of oxygen uptake in the initial recovery phase. This observation is additionally supported by the high positive correlation of the parameter B with T_1/2_ (*r* = 0.61, *p* = 0.016). We have also observed high positive correlation (*r* = 0.77, *p* = 9 × 10^–4^) between relative VO_2__peak_ and C parameter which is related to oxygen uptake value in the final stage of the REC phase.

According to Table 2 a positive Pearson correlation (*r* = 0.45, *p* = 0.223) was observed between the Sm_max_ parameter and relative VO_2__peak_. Negative correlation −0.36 between the Hill’s coefficient n and the individualized VO_2__peak_ was also obtained (however, both not significant, *p* = 0.304). We did not obtain correlation between k parameter and classical CPET measures. In the last part of column (Table 2), we considered median values of percentage oxyhemoglobin level from BAS, RecBegin, and RecEnd phases.

The smaller the percentage differences in saturation between BAS and RecEnd indicates ability to faster return to the resting state. The negative correlation between n and RecEnd allows to conclude that higher n parameter indicates easier oxygen binding capacity and thus lower difference between the mean BAS and RecEnd saturation. The positive high correlation between the constant k and RecEnd is also related to the presented interpretation. The shorter time needed to return to 50% of Sm, the smaller difference between BAS and RecEnd.

## Discussion

The main purpose of this study was to propose two non-linear models allowing quantitative analysis of the recovery phase in the incremental protocol. We hypothesized the usefulness of these tools in the assessment of the respiratory and muscular responses to the exercise in healthy subjects. It was verified by the analysis of the correlation between model parameters and markers reflecting aerobic capacity. A positive correlation between k parameter and median of oxyhemoglobin during RecEnd indicates, that the time needed to return to half the Sm_max_ is related to the median post-exercise oxyhemoglobin concentration value. Additionally, the Sm_max_ coefficient from the Hill’s model specifying the maximal post-exercise oxyhemoglobin values correlates positively with VO_2__peak_. We observed a high correlation between VO_2__peak_ and T_1/2_ as well as n with Median of Sm (%) in RecEnd, possibly evidencing peripheral oxygen binding kinetics being one crucial aspect of aerobic capacity. Simultaneously, the process of short-term recovery is characterized by the fast decrease of oxygen uptake. The negative correlation of n with Sm (%) in RecEnd indicates increased oxygenation of hemoglobin. During the fast decrease of oxygen uptake the hemoglobin affinity to oxygen progressively declines due to the decreasing number of free hemoglobin binding sites. This saturation causes a plateauing of Sm (%) in recovery (compare Figure 2B and Chang et al., 2020).

VO_2max_ is a popular index to assess the capacity of the cardiovascular-metabolic system. However, conclusions based on this indicator may be difficult due to the requirement of maximum effort from patients who could be not adjust to intense exercise (Cohen-Solal et al., 1995). Thus, in clinical practice VO_2peak_ is assessed and also considered for correlation determination in this study. A better understanding of the VO_2_ kinetics, especially its relationship to the plasticity of the oxygen transporting and cooperating systems (the muscular system during exercise), is important for improving training adaptations not only in athletic populations, but primarily health in patients suffering from depressed VO_2_ kinetics and in the growing elderly population (Cohen-Solal et al., 1995).

The conclusion presented in this paper allow to summarize that the problem of assessing adaptation to physical effort on the basis of measures obtained from the REC is complex and therefore not easy to solve. The specificity of the recorded data related to short CPET signals was an undoubted limitation in the choice of methods proposed here for the quantitative description of the aerobic capacity of participants. Bearing in mind the above limitations, a dedicated approach was developed to assess suitable response to physical effort on the basis of the REC phase using two models. An exponential model was used to describe the kinetics of changes in oxygen uptake and Hill model for oxyhemoglobin percentage concentration during REC phase. The physiological interpretation of the estimated coefficients was performed and analysis of correlation with the classic measures of physical endurance from the CPET test during recovery phase.

The pattern of oxygen consumption kinetics during recovery has been analyzed in recent studies (Cohen-Solal et al., 1995; Vianna et al., 2014). The work (Cohen-Solal et al., 1995) proposes the analysis of the kinetics of oxygen uptake during rest after maximum graded exercise in patients with chronic heart failure (CHF). The hypothesis put forward by the authors of the paper, indicates that the kinetics of the return of oxygen uptake to the resting phase after exercise is prolonged in parallel with the recovery of muscle energy reserves, and the recovery time does not depend on the intensity of the maximum load. In the context of this study, the above conclusions about the promising prognostic value of the T_1/2_ parameter seem to justify the attempt to confront the rate of recovery of the oxygen uptake level in the group of healthy young subjects with the traditional CPET indicators. In the second study (Vianna et al., 2014), the exponential model was used to analyse the rate of recovery of oxygen uptake in a group of 14 bodybuilders after performing one of 4 randomly applied intense strength training. In referred approach, the analogous formula to Eq. (1) (also for the HR signal) was used to determine the time needed to reach 63% of VO_2__peak_. The study reports that in healthy and training people, VO_2_ kinetics depends on muscle metabolism and is not limited to a large extent by the efficiency of O_2_ transporting systems. On the other hand, aging may be a factor that changes the control of VO_2_ kinetics from the muscular system to the transport system associated with circulating hemoglobin (Vianna et al., 2014). The above conclusion may explain our relatively low correlation values between Hill’s model and VO_2__max_ and VO_2__peak_ parameters.

The topic of changes in metabolic functions of skeletal muscles caused by exercise has already been discussed (Hargreaves and Spriet, 2020; Thyfault and Bergouignan, 2020). The muscle oxygenation recovery, characterized by markers obtained from oxyhemoglobin concentration signal, and its relationship with aerobic capacity and aerobic powerare considered in research studies. However, developing non-invasive techniques that quantify the degree of metabolic adaptation and identify the complex responses of the human body to exercise is an ongoing challenge for future research. Kinetics of oxyhemoglobin in the recovery phase has been explored by Ferreira et al. (2005). The authors proposed a mathematical model, which was fitted to the changes in oxyhemoglobin and total hemoglobin concentration during the recovery phase. The fitting parameters were then used to quantify the blood flow kinetics of light and vigorous exercise. In Stöcker et al. (2017) authors investigate the effect of six different intensities on relative changes oxyhemoglobin concentration during recovery phase as an indicator for microvascular O_2_ distribution in healthy active and non-athletes male group. Results demonstrate an overshoot in oxyhemoglobin concentration following exercise at 80% VO_2__peak_ compared with exercise at 40% VO_2__peak_, where highest post-exercise values are reached at the end of the 5-min recovery period. In the proposal of the Hill’s model for assessment of the changes in the concentration of oxyhemoglobin, we expected a correlation between model coefficients and the aerobic capacity. In accordance to Stöcker et al. (2017), we also obtained a relationship between the final 5 min of the recovery phase (RecEnd) and the model coefficients.

This study was limited by the small and homogeneous sample and the all-out protocol. It should also be mentioned that the NIRS device accounts not only for hemoglobin but also myoglobin. Further studies should be performed to compare our findings with other type of protocols and subjects.

In conclusion, in this paper two different models have been proposed for the analysis of the oxygen uptake and oxyhemoglobin concentration signals during the recovery phase. We hypothesized the usefulness of these methods in the assessment of the respiratory and muscular responses to the exhaustive exercise in healthy people. For this purpose the physiological interpretation of the estimated coefficients was made and they were correlated with the traditional measures of aerobic capacity from the CPET test. High correlations indicate the usefulness of these markers in the assessment of the recovery process in healthy subjects under certain submaximal conditions. Additionally, our approach can be considered as a proof of concept correlating oxygen binding kinetics during such submaximal workload conditions, i.e., recovery at 50 W cycling in our study, with aerobic capacity and training status. In the study, we used non-invasive, easy applicable NIRS-monitoring of working muscle oxygenation kinetics during short term the recovery. However, developing non-invasive techniques that quantify the degree of metabolic adaptation and identify the complex responses of the human body to exercise is an ongoing challenge for future research. In next studies, standardized submaximal test protocols should be evaluated in terms of practical applicability of this concept.

## Data Availability Statement

The raw data supporting the conclusions of this article will be made available by the authors, without undue reservation.

## Ethics Statement

The studies involving human participants were reviewed and approved by the local ethics committee at the University of Rostock. The patients/participants provided their written informed consent to participate in this study.

## Author Contributions

MW and MP designed and performed the experiments. MP and MŻ were responsible for methodology of calculations. MŻ did the data analysis and prepared results presentation. MW, MP, and MŻ interpreted the results and wrote the manuscript. All authors contributed to the article and approved the submitted version.

## Conflict of Interest

The authors declare that the research was conducted in the absence of any commercial or financial relationships that could be construed as a potential conflict of interest.
